# Human melioidosis reported by ProMED

**DOI:** 10.1016/j.ijid.2015.05.009

**Published:** 2015-06

**Authors:** Katherinn Melissa Nasner-Posso, Stefania Cruz-Calderón, Franco E. Montúfar-Andrade, David A.B. Dance, Alfonso J. Rodriguez-Morales

**Affiliations:** a Public Health and Infection Research Group and Incubator, Faculty of Health Sciences, Universidad Tecnológica de Pereira, Pereira, Risaralda, Colombia; b Infectious Disease Section, Department of Internal Medicine, Hospital Pablo Tobón Uribe, Medellín, Antioquia, Colombia; c Lao–Oxford–Mahosot Hospital Wellcome Trust Research Unit, Vientiane, Lao People's Democratic Republic; d Centre for Tropical Medicine and Global Health, University of Oxford, Oxford, UK; e Working Group on Zoonoses, International Society for Chemotherapy, Aberdeen, UK; f Committee on Zoonoses and Haemorrhagic Fevers, Asociación Colombiana de Infectología, Bogotá, DC, Colombia

**Keywords:** Melioidosis, ProMED, Epidemiology

## Abstract

**Objective:**

There are limited sources describing the global burden of emerging diseases. A review of human melioidosis reported by ProMED was performed and the reliability of the data retrieved assessed in comparison to published reports. The effectiveness of ProMED was evaluated as a source of epidemiological data by focusing on melioidosis.

**Methods:**

Using the keyword ‘melioidosis’ in the ProMED search engine, all of the information from the reports and collected data was reviewed using a structured form, including the year, country, gender, occupation, number of infected individuals, and number of fatal cases.

**Results:**

One hundred and twenty-four entries reported between January 1995 and October 2014 were identified. A total of 4630 cases were reported, with death reported in 505 cases, suggesting a misleadingly low overall case fatality rate (CFR) of 11%. Of 20 cases for which the gender was reported, 12 (60%) were male. Most of the cases were reported from Australia, Thailand, Singapore, Vietnam, and Malaysia, with sporadic reports from other countries.

**Conclusions:**

Internet-based reporting systems such as ProMED are useful to gather information and synthesize knowledge on emerging infections. Although certain areas need to be improved, ProMED provided good information about melioidosis.

## Highlights

• ProMED would be used to assess the reliability of the data of emerging infectious diseases, such as melioidosis.
• The study evaluated the effectiveness of ProMED as a source of epidemiological data by focusing on melioidosis.
• This work identified 4630 cases of melioidosis with an overall case fatality rate of 11%, mainly reported from Australia, Thailand, Singapore, Vietnam, and Malaysia.
• Although certain areas need to be improved, ProMED provided good information about melioidosis.

## 1 Introduction

Melioidosis is a potentially fatal tropical disease caused by a Gram-negative bacillus called *Burkholderia pseudomallei*.^1–3^ This disease is acquired by inhalation, inoculation, or ingestion of microorganisms normally present in water and moist soil,^4^ and more rarely from other infected individuals (e.g., mothers with mastitis).^1^ Melioidosis is known to be endemic in Southeast Asia and Northern Australia, particularly between latitudes 20° N and 20° S, however sporadic cases have been reported in the Caribbean, Central America, and South America.^5,6^ The incidence increases at times of heavy rains, and the population groups at greatest risk are farmers and indigenous inhabitants of rural tropical areas.^4,7^ The disease usually has an incubation period of 1 to 21 days, although long periods of latency may occur, and has a mortality rate approaching 40% in developing countries.^8,9^ It causes a wide spectrum of clinical manifestations^10^ that includes asymptomatic seroconversion, pneumonia, atypical forms with neurological involvement, and severe forms causing septicaemia and death.^8,11^ Alcohol consumption, diabetes mellitus, and chronic kidney disease are risk factors for more severe disease.^1,11^ *B. pseudomallei* infection requires prolonged antibiotic therapy to achieve the eradication of the organism and prevent relapses.^12^

The global burden of melioidosis needs to be determined, but there are limited sources from which to retrieve data regarding the disease, which is not statutorily notifiable in most countries. ProMED (The Program for Monitoring Emerging Diseases) is an internet-based reporting system for emerging infectious or toxin-mediated diseases.^13–15^ It was founded in 1994 and is currently a program of the International Society for Infectious Diseases.^13–16^ Its mission is to serve global health by the immediate dissemination of information that may lead to early preventive measures, prevent the spread of outbreaks, and provide more accurate disease control. Its sources of information include media reports, official reports, online summaries, reports from local observers, and others.^16^ The reports are reviewed and edited by expert moderators in the field of infectious diseases and are then submitted to the mail server and published on the website and disseminated to subscribers by e-mail. Being a non-profit, non-governmental system, it is free from governmental constraints.^13–16^ It currently has over 70 000 subscribers in over 185 countries.^16^

In this study, the human melioidosis incidents reported by ProMED were reviewed and the reliability of the data retrieved assessed in comparison to published reports. The effectiveness of ProMED as an epidemiological data source was thus evaluated by focusing on melioidosis.

## 2 Methods

The keyword ‘melioidosis’ was used in the ProMED search engine, looking at ProMED-mail (English), ProMED-ESP (Spanish), ProMED-FRA (French), and ProMED-PORT (Portuguese). All the information in the individual reports was reviewed and the data collected using a structured form, including year, country, gender, occupation, the number of infected individuals, and the number of fatal cases.

## 3 Results

One hundred and twenty-four entries reported between January 1995 and October 2014 were identified (119 from ProMED-mail, four from ProMED-ESP, and one from ProMED-PORT). A total of 4630 cases were reported, with 505 cases recorded as being fatal, giving a reported case fatality rate (CFR) of 11%. Gender was only recorded in 20 cases, with 12 (60%) of these being male. Of 17 cases for which the age was reported, the median aged was 45 years (range 17–80 years). The occupational status was only reported for six, of whom three were farmers, one was a gardener, one was a housewife, and one was a student. Twenty-five cases were reported as imported. Countries of origin were India (*n* = 9; all to the UK), Thailand (*n* = 5; three cases to Finland, one to Belgium, and one to Israel), Bangladesh (*n* = 3; all to the UK), Honduras (*n* = 2; both to the USA), Gambia (*n* = 1; to Spain), and Madagascar (*n* = 1; to Belgium); two cases had travelled to Finland and Spain from unspecified countries in Asia and Africa, respectively.

The distribution of the infected and fatal cases by country is presented in Table 1. Most of the cases were reported from Australia, followed by Thailand, Singapore, Vietnam, and Malaysia. The lowest fatality rates were reported from Australia (13%) and Malaysia (15%). Amongst cases from Australia, 1381 (83%) were from the Northern Territory (with at least 1059 from Darwin (77%)).

## 4 Discussion

It is well known that *B. pseudomallei* infection is endemic in Southeast Asia and Northern Australia, but as sporadic cases have been reported with a wide geographical distribution, systems such as ProMED provide an opportunity to alert the international community to the occurrence of cases around the world, including the identification of new foci of endemicity. In addition, the occurrence of clusters of cases in an atypical location might alert public health authorities to the possibility of the deliberate release of *B. pseudomallei*, a tier 1 biothreat agent.^17^ When compared with the information provided by reports in the subject literature, the most notable finding was that the CFR identifiable from ProMED was low in comparison with the published literature from the developing world;^6,8,18,19^ however, under-reporting of fatal cases by ProMED is not surprising, as the purpose is to act as an early alert system, and outcomes may not be known at the time of posting. In addition, the data were very incomplete with regards to characteristics such as gender, age, and occupation.

In the present study, the highest numbers of reported cases of melioidosis on ProMED were from Australia (1664 cases) and Thailand (1314), which is consistent with the known epidemiology of the disease, although the total number of cases diagnosed each year in Thailand is higher than that in Northern Australia due to the greater numbers of exposed individuals.^5,6,18–21^ Countries like Singapore and Malaysia are also known to be endemic for melioidosis,^5^ whilst Taiwan, Brazil, and Papua New Guinea have also reported sporadic cases of melioidosis, sometimes related to specific climate events,^7,22–24^ which is also consistent with the results found in ProMED.

Within Australia, melioidosis is most frequently reported from north Queensland and the ‘Top End’ of the Northern Territory. One hundred and seventy-six cases confirmed by culture were reported from northern Queensland during the period from 2000 to 2009, with an overall CFR of 21%, falling to 14% in the years between 2005 and 2009.^25,26^ A similar CFR (19%) was reported from the Northern Territory during the years 1990–2000,^26,27^ comparable to the 13% mortality amongst cases reported on ProMED (Table 1). The greater representation of reports from the Northern Territory of Australia on ProMED is likely to be skewed, reflecting the fact that the public health authorities in the Northern Territory have a proactive annual publicity campaign to alert people to the risk of melioidosis at the start of the rainy season, which is then picked up by ProMED.

In Singapore, the mortality rate decreased from a CFR of 60% in 1989 to 27% in 1996,^28^ with 693 cases of melioidosis and 112 deaths reported between 1998 and 2007, equivalent to a CFR of 16.2%,^29^ which is also close to that found from ProMED (24%) (Table 1). Between 2003 and 2012, 550 cases were reported in Singapore, with rainfall and humidity levels being found to be associated with disease incidence during that period.^20^

The relatively low CFR in developed countries such as Singapore and Australia is likely to be related to increased awareness amongst medical staff, allowing earlier diagnosis and treatment of early-stage disease,^28^ optimal antibiotic therapy, and improved supportive management. On the other hand, a prospective cohort study in the northeast of Thailand identified 2243 patients between 1997 and 2006, with a CFR of 42.6% (the third leading cause of death from infectious diseases after HIV and tuberculosis),^20^ a rate that is much higher than the data from ProMED would suggest (Table 1).

By contrast, although ProMED captured information about some countries in which melioidosis is rarely reported, such as Madagascar, Mauritius, and Brazil, the high CFRs (100%, 100%, and 80% respectively; Table 1) amongst cases from these countries reported on ProMED are likely to be due to the low denominators, with reporting skewed to the recognition of more severe cases, compounded by a lack of awareness amongst medical staff leading to sub-optimal case management. Clearly ProMED cannot always be regarded as an accurate source of information about patient outcome, which is not surprising as that is not its purpose.

For Vietnam, the 343 cases included in Table 1 corresponded to historic information reported to ProMED-mail from GIDEON (http://www.gideononline.com/), a system that captures and collates information about infectious diseases from a wide range of sources including the published literature, and related to cases that were reported (as of 1973) among American Troops during the Vietnam War. The disease is probably still prevalent in Vietnam, although it is relatively rarely diagnosed among the indigenous population.

While this search on ProMED identified no cases reported from Colombia, indigenous melioidosis has recently been recognized there, with 10 cases originating from different parts of the country recently reported in the Spanish literature.^30,31^ Indeed, Brazil is the only country in the Americas amongst the ProMED reports, despite the fact that sporadic cases have also been reported from other countries in the region, such as Costa Rica and Puerto Rico.^9,32,33^ It is likely that the low reported incidence of melioidosis in countries of the Americas is associated with a lack of awareness amongst clinical and laboratory staff, leading to a failure to identify the microorganism, thereby underestimating the true incidence of infection.^20,29^ It is important to ensure that the identification of melioidosis in a new area is widely communicated to healthcare staff in order for appropriate methods to be used to confirm the diagnosis by sampling, laboratory culture, isolation, and identification of *B. pseudomallei* whenever possible.^8^ Furthermore, it is notable that more than 95% of ProMED reports were in English and less than 5% in other languages, including Spanish, meaning that those who only subscribe in languages other than English would not have received significant information about melioidosis. This has probably been influenced by the fact that there is not yet enough awareness of melioidosis in Francophone and Hispanophone countries in Latin American, the Caribbean, and Africa.

Although ProMED proved to be a useful source of information about melioidosis, it was evident that it gave a misleading impression of the distribution and mortality in some areas, as has been reported previously for other infections.^13^ We think it would be useful to consider the adoption of standardized templates for reporting to ProMED in order to enhance the standardization of data and provide clear, accurate, and reliable information that could be used to conduct epidemiological analyses in order to establish the status of emerging diseases and assist in their recognition and control. In addition, we think it would be useful for reports in one language to be mirrored to ProMED in other languages, especially when it is the language of the country from which the report originated.

In addition, ProMED may be useful as a source of information for travel medicine practice.^10^ Melioidosis is well described amongst both short- and long-term visitors to endemic areas and so needs to be considered in the evaluation and treatment of patients with sepsis or other febrile illnesses returning from endemic countries,^34,35^ particularly from Southeast Asia and Northern Australia as has been seen in this study. Melioidosis should be considered in anyone with compatible clinical manifestations, even in the absence of apparent risk of exposure to *B. pseudomallei*. A greater appreciation of this disease among physicians in non-endemic areas should lead to better management of imported cases, and ProMED could also be a source of epidemiological information when giving pre-travel advice and conducting post-travel consultations.

In regions, such as South America, where reports of melioidosis have emerged relatively recently, strategies for surveillance and recognition of melioidosis need to be strengthened. Working groups of national infectious diseases societies could help to increase the awareness among general and infectious diseases practitioners, but might also influence public health authorities by suggesting that melioidosis becomes a notifiable disease in each country.

In conclusion, information systems such as ProMED provide insight through daily reports of the occurrence of both individual cases and clusters of infectious diseases. This helps the rapid dissemination of knowledge of the current global situation of emerging infectious diseases and their outcomes in terms of morbidity, disability, and death. However, the data on ProMED are often incomplete and it is important to continue improving and supplementing these systems to allow a more precise knowledge of the worldwide epidemiology of emerging diseases such as melioidosis.

*Funding*: Universidad Tecnologica de Pereira, Pereira, Risaralda, Colombia.

*Conflict of interest:* There is no conflict of interest.

*Contributions:* Study design: AJRM; data collection: KMNP, SCC, AJRM; data analysis: KMNP, SCC, AJRM; writing: all authors. All authors read the final version submitted.

## Figures and Tables

**Table 1:** The number of infected and fatal melioidosis cases reported to ProMED between 1995 and 2014

Country	Infected patients, *n*	Reported deaths, *n*	CFR (%)
Australia	1664	217	13
Thailand	1314	0	0
Singapore	941	227	24
Vietnam	343	0	0
Malaysia	221	34	15
Taiwan	77	12	16
UK^a^	14	0	0
India	12	0	0
Brazil	10	8	80
Finland^a^	6	0	0
Papua New Guinea	6	3	50
Myanmar	6	0	0
Bangladesh	5	0	0
Belgium^a^	2	0	0
Spain^a^	2	0	0
USA^a^	2	1	50
Madagascar	2	2	100
Israel^a^	1	0	0
Mauritius	1	1	100
Sri Lanka	1	0	0
Total	4630	505	11

CFR, case fatality rate.

a Imported cases.
